# A Novel Approach to Enhancing Ganoderic Acid Production by *Ganoderma lucidum* Using Apoptosis Induction

**DOI:** 10.1371/journal.pone.0053616

**Published:** 2013-01-10

**Authors:** Bang-Jau You, Miin-Huey Lee, Ni Tien, Meng-Shiou Lee, Hui-Chuan Hsieh, Lin-Hsien Tseng, Yu-Lin Chung, Hong-Zin Lee

**Affiliations:** 1 Department of Chinese Pharmaceutical Sciences and Chinese Medicine Resources, China Medical University, Taichung, Taiwan; 2 Department of Plant Pathology, National Chung-Hsing University, Taichung, Taiwan; 3 Department of Laboratory Medicine, China Medical University Hospital, Taichung, Taiwan; 4 School of Pharmacy, China Medical University, Taichung, Taiwan; University of Wisconsin – Madison, United States of America

## Abstract

*Ganoderma lucidum* is one of most widely used herbal medicine and functional food in Asia, and ganoderic acids (GAs) are its active ingredients. Regulation of GA biosynthesis and enhancing GA production are critical to using *G. lucidum* as a medicine. However, regulation of GA biosynthesis by various signaling remains poorly understood. This study investigated the role of apoptosis signaling on GA biosynthesis and presented a novel approach, namely apoptosis induction, to increasing GA production. Aspirin was able to induce cell apoptosis in *G. lucidum*, which was identified by terminal deoxynucleotidyl transferase mediated dUPT nick end labeling assay positive staining and a condensed nuclear morphology. The maximum induction of lanosta-7,9(11), 24-trien-3α-01-26-oic acid (ganoderic acid 24, GA24) production and total GA production by aspirin were 2.7-fold and 2.8-fold, respectively, after 1 day. Significantly lower levels of GA 24 and total GAs were obtained after regular fungal culture for 1.5 months. ROS accumulation and phosphorylation of Hog-1 kinase, a putative homolog of MAPK p38 in mammals, occurred after aspirin treatment indicating that both factors may be involved in GA biosynthetic regulation. However, aspirin also reduced expression of the squalene synthase and lanosterol synthase coding genes, suggesting that these genes are not critical for GA induction. To the best of our knowledge, this is the first report showing that GA biosynthesis is linked to fungal apoptosis and provides a new approach to enhancing secondary metabolite production in fungi.

## Introduction

*Ganoderma lucidum* is a basidiomycete fungus and has been one of mostly widely used folk remedy in Asia for thousands years. Patients with diseases such as cancer, chronic hepatitis, inflammation, hypertension, and heart disease are treated with *G. lucidum* [1]. Moreover, the fungal mycelium of *G. lucidum* is consumed as a tonic to delay senility, improve immunity and enhance health in Taiwan. Ganoderic acids (GAs) are one of major compounds with pharmacological activity found in *G. lucidum* and these compounds belong to the triterpenoids. More than 130 triterpenoids have been isolated and characterized from the fruiting bodies, cultured mycelium and spores of *G. lucidum* [1], [2]. Ganoderic acids from *G. lucidum* have been shown to have numerous biological activities including anticancer activity, antiviral activity, hepatoprotective effects, anti-platelet aggregation effects, anti-oxidant activity, hypocholesterolemic activity, and the inhibition of histamine release [1], [3]. Recently, ganoderic acid T has been demonstrated to inhibit tumor metastasis by suppression of NF-κB activation [4]. P53 also play important role for anti-invasion of ganoderic acid T in cancer cell [5]. Moreover, several studies indicated that mitochondria and p53 may be targeted by ganoderic acid T and Me to induce cell apoptosis [6]–[8].

The biosynthesis of triterpenoids has been proposed to proceed via the mevalonate/isoprenoid pathway. Acetyl CoA is used to synthesize mevalonate and isopentenyl-pyrophosphate, which subsequently becomes farnesyl diphosphate [9], [10]. Squalene synthase (SQS) and lanosterol synthase (LS) have been proposed to be involved in the formation of squalene and lanosterol, respectively [11], [12]. Biosynthesis of the GA end products are thought to be synthesized from lanosterol by a series of oxidation, reduction, hydroxylation, and acetylation steps [3].

Due to the long cultivation time needed to produce fruiting bodies, intensive studies have targeted improving the production of fungal biomass and GAs in submerged culture [13], [14]. The application of various inducers, such as phenobarbital and methyl jasmonate, has been used to enhance GA production in submerged culture [15], [16]. Our recent studies have revealed that *G. lucidum* produces large quantity of GAs when cultured on solid-state medium [17]. However, regulation of triterpenoids biosynthesis and its signal transduction remains enigmatic for *G. lucidum*. Only a few studies have been carried out and these have suggested that calcium and reactive oxygen species (ROS) are involved in the regulation of GA biosynthesis [18]–[20]. The characterization of GA biosynthetic regulation would be valuable and might help to enhance GA production, which would be important to the functional food and pharmacological industries.

Apoptosis in fungi is an emerging field and is less well developed than the corresponding studies in mammals. In yeast, the physiological roles of apoptosis have been shown to include the control of the replicative life-span and to affect the long-term survival of yeast colonies [21], [22]. High concentrations of yeast pheromones, heterologous expression of pro-apoptotic genes, defects in cellular processes, and exogenous stress, which includes H_2_O_2_, acetic acid, and UV radiation, are able to induce yeast apoptosis [21]–[23]. Aspirin has also been shown to induce apoptosis in yeast and mammalian cells [24], [25]. However, to the best of our knowledge, the regulation of secondary metabolite biosynthesis by apoptosis signaling has never been studied in fungi.

A previous study by us showed that a high dose of H_2_O_2_ or the pro-oxidant 1-chloro-2,4-dinitrobenzene (CDNB) is able to induce GA production and in the process reduces fungal biomass. However, incubating *G. lucidum* with low doses of H_2_O_2_ and CDNB has no effect on GA and biomass production [19]. These results suggest that cell death is related to GA production in *G. lucidum*. Therefore, in this study, we have tested the hypothesis that apoptosis signaling is linked to GA biosynthesis. A further aim of this study was to apply this novel approach, the induction cell apoptosis, to enhancing the production of fungal secondary metabolites. In the current study, the fungal mycelium of *G. lucidum* was incubated with aspirin and cell apoptosis was evaluated. GA production and the expression of genes involved in the biosynthesis of GAs were measured. Important regulators of cell apoptosis, including ROS production and MAPK phosphorylation, were also examined to evaluate their putative roles in apoptosis and GA biosynthesis.

## Materials and Methods

### The fungal strain and its culture conditions

The BCRC 36111 strain of *Ganoderma lucidum* was purchased from the Bioresource Collection and Research Center (Hsin Chu, Taiwan). The fungus was maintained on potato dextrose agar (PDA; Difco, Sparks, MD, USA) plate at 28°C. Fungal mycelium grown on PDA overlaid with a layer of cellophane for 7 to 10 days at 28°C was used as inoculum. To test the effect of aspirin on GA production and biomass production, fungal mycelium (8.75 g) obtained from a 7–10 day-old culture was dispersed in sterile water (50 mL) using a sterile blender. Fungal mycelium of 70 mg was spread onto PDA (9-cm diameter petri dish) with a layer of sterile cellophane for 4–12 days at 28°C. Fungal mycelium was then transferred to 25-mL of PDB in a 250 mL flask and treated with aspirin for 6 to 48 hr with shaking (100 rpm) at 28°C. Fungal mycelium was then harvested, dried, weighted, and subjected for GA extraction. To evaluate GA and biomass production of fungal culture on PDA, fungal mycelium of 70 mg was applied to PDA with a layer of sterile cellophane for 1 to 6 weeks at 28°C. Fungal mycelium was then peeled from the cellophane layer to determine biomass and GA production. All treatments were carried using at least three replicates and were repeated at least 3 times.

### TUNEL assay and nuclear staining

Terminal deoxynucleotidyl transferase mediated dUPT nick end labeling (TUNEL) assays were carried out using the *In Situ* Death Detection kit (Roche Applied Science, Indianapolis, IN, USA). Fungal cells of *G. lucidum* were treated with aspirin for 16 hr, fixed with 4% paraformadehyde for 1 hr, washed with PBS, and then digested with cell wall degrading enzymes (0.5 U mL^−1^ of driselase, 1050 U mL^−1^ of β-glucaronidase, 81.25 U mL^−1^ of lyticase, 5 mg mL^−1^ of lysing enzyme, and 0.015 U mL^−1^ of chitinase) for 30 min. Cell permeabilization followed by the TUNEL reaction were conducted according to the manufacturer's guidelines. To obtain a control nuclear morphology for normal cells using TUNEL staining, fungal cells were incubated with 0.235 U µL^−1^ of DNase I after cell permeabilization and then assessed using the TUNEL reaction mixture. After TUNEL staining, the fungal cells were incubated with 2 µg mL^−1^ of 4′, 6-diamidino-2-phenylindole (DAPI) in 75% ethanol for 1 hr to allow examination of the cell nuclei. Photographs were taken by fluorescence microscopy (IX70, Olympus, Tokyo, Japan). At least three independent experiments were performed.

### Detection and quantification of ganoderic acids by HPLC

Extraction and detection of ganoderic acids (GAs) from the fungal mycelium was performed as previously described [17]. Dried mycelium (100 mg) was extracted with methanol, and GAs in supernatant was analyzed by HPLC. Lanosta-7,9(11), 24-trien-3α-o1-26-oic acid (ganoderic acid 24) was used to construct a calibration curve for production of GA24 and total GAs in the fungal mycelium [17]. Total GAs produced by the fungal mycelium was calculated by adding the peak areas of compounds eluted from 5 to 50 min by HPLC analysis [17].

### Expression of the genes encoding a squalene synthase and a lanosterol synthase

DNA fragments encoding a putative squalene synthase (SQS) and a putative lanosterol synthase (LS) were amplified from *G. lucidum* BCRC 36111 [17]. Northern blotting analysis was performed using standard procedures. Fungal total RNA was extracted by Trizol reagent (Invitrogen, Carlsbad, CA, USA). Digoxigenin-11-dUTP (Roche Applied Science) was incorporated into the SQS DNA by PCR using the primers glssF263 (5′TGGACACGATCGAAGATGACATGAC3′) and glssR1492 (5′GCCATCGTTTGTGGGATCGCACAGAA3′). A DNA probe specifically hybridizing to the LS sequence was amplified in a similar way using the primers gllsF1292 (5′CGGCGTATCGGCACCAGACGAA3′) and gllsR2105 (5′TTCGGGTACGATATCGCGACGTTC3′). Immunological detection of the Northern Blots using CDP-Star chemiluminescent substrate was conducted following the manufacturer's recommended procedures (Roche Applied Science). All experiments were conducted at least three times.

### Detection of ROS generation

The accumulation of reactive oxygen species (ROS) in fungal cells was detected by 2′,7′-dichlorofluorescin diacetate (DCFH-DA). Fungal mycelium that had been cultured on PDA for 2 days was pre-treated with 10 µM DCFH-DA for 1 hr in H_2_O. Aspirin at concentrations ranging from 1 mM to 8 mM was then incubated with mycelium for 4 hr. Photographs were taken by fluorescence microscopy (IX70, Olympus, Tokyo, Japan ; excitation 480 nm, emission 520 nm). To quantify ROS accumulation, the fluorescence was detected using Bioteck synergy multidetection microplate reader with excitation wavelength at 485 nm and emission wavelength at 528 nm. At least three independent experiments were conducted.

### Assay detecting Hog1 MAPK phosphorylation

Fungal proteins were extracted from mycelium using extraction buffer (50% glycerol, 20 mM Tris-HCl, pH 7.5, 1 mM EDTA, 0.1 mM DTT, 50 mM NaF, 0.2 mM PMSF, 5 mM Na_3_VO_4_). After centrifugation (22,000× g) for 20 min, the concentration of proteins was quantified by Quick Start Bradford Protein Assay (Bio-Rad, Hercules, CA, USA). Proteins were separated on a denaturing 10% SDS-polyacrylamide gel and then transferred onto Polyscreen PVDF transfer membrane (Perkin Elmer, Waltham, MA, USA). Anti-phospho-p38 MAPK (Thr180/Tyr182) rabbit monoclonal antibody (Cell Signaling Technology, Beverly, MA, USA) were used as the primary antibody to detect phosphorylation of Hog-1. The intensity of β actin detected using mouse monoclonal anti-beta actin antibody (Abcam, Cambridge, MA, USA) acted as the loading control. HRP-conjugated goat anti-rabbit IgG (KPL, Gaithersburg, MD, USA) or a rabbit anti-mouse IgG (GeneTex, Irvin, CA, USA) was used as secondary antibodies as appropriate. Hybridization of membrane with the antibodies was performed according to the manufacturer's guidelines. The signals were detected using Immobilon Western Chemiluminescent HRP Substrate (Millipore, Billerica, MA, USA). All experiments were performed at least three times.

### Statistical analysis

The statistical analysis of GA production and biomass production across the various different treatments was carried out by Student's t-test (Microsoft Excel, Seattle, WA, USA). Statistical significance was expressed as ^*^ *p*<0.05, ^**^ *p*<0.01, ^***^ *p*<0.001.

## Results and Discussion

### Effect of aspirin on the production of ganoderic acids and fungal biomass

**Figure 1:**
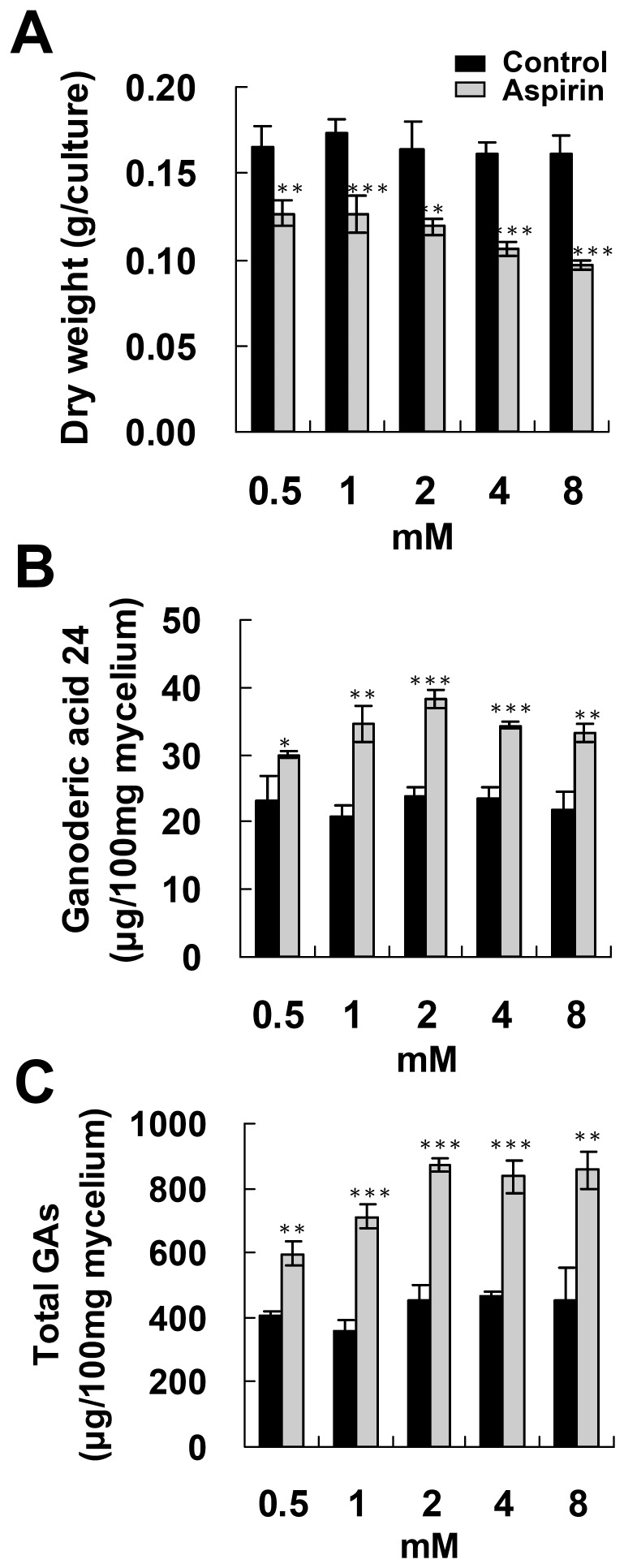
Effect of aspirin concentration on the accumulation of ganoderic acids and fungal biomass. Fungal mycelium was cultured on PDA for 4 days and then incubated with 0.5–8 mM aspirin for additional 1 day. Fungal biomass (A), accumulation of lanosta-7,9(11), 24-trien-3α-o1-26-oic acid (ganoderic acid 24) (B) and total ganoderic acids (total GAs) (C) were evaluated. The means of three independent samples with standard deviations are presented. *p<0.05, **p<0.01, ***p<0.001 as compared with the control group.

To evaluate the effect of aspirin on ganoderic acid production, BCRC 36111 was cultured on PDA for 4 days and then incubated with 0.5–8 mM aspirin for 1 day. Biomass production was gradually reduced as the aspirin concentration increased (Figure 1A). Incubation with 0.5 mM aspirin slightly increased total GAs production and lanosta-7,9(11), 24-trien-3α-o1-26-oic acid (ganoderic acid 24, GA 24) production. Higher doses of aspirin (1–8 mM) significantly increased GA production (Figure 1B and 1C).

**Figure 2:**
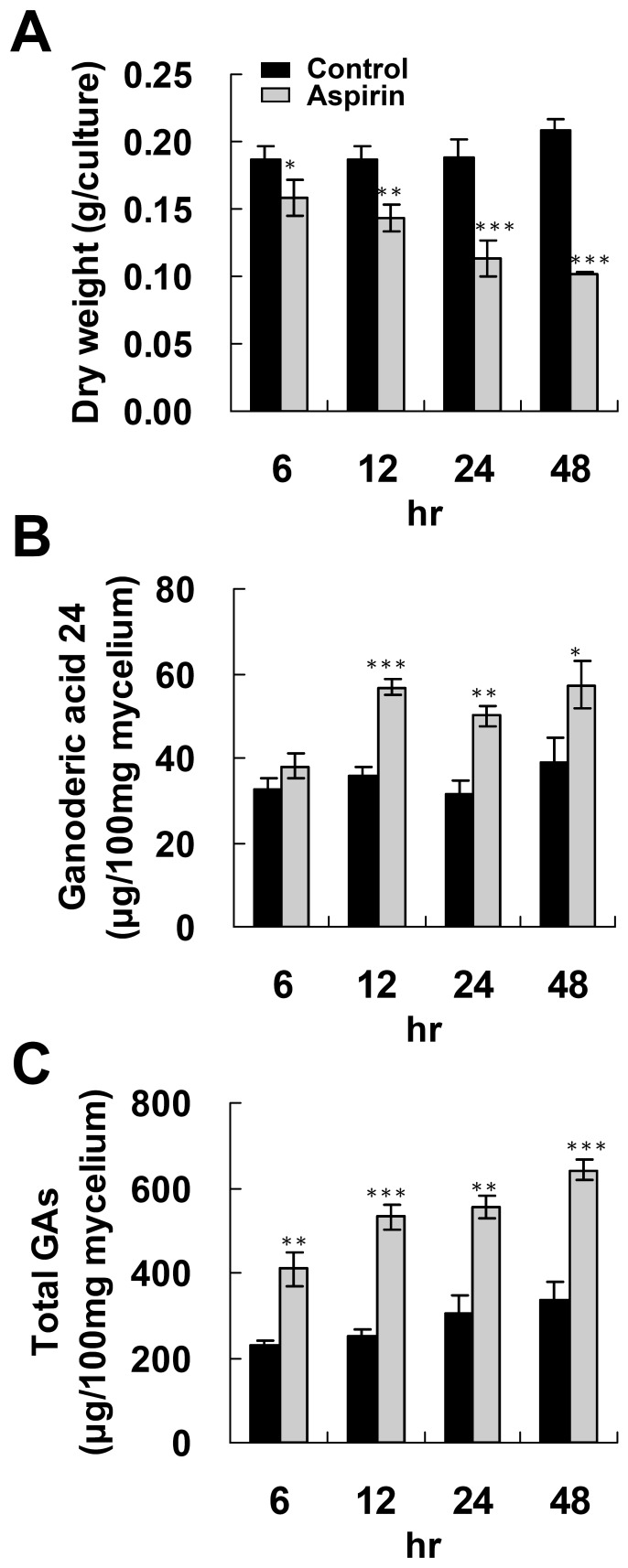
Time course of ganoderic acids and fungal biomass production of *Ganoderma lucidum* incubated with aspirin. Fungal mycelium was cultured on PDA for 4 days and then incubated with 4 mM aspirin for additional 6 to 48 hr. Fungal biomass (A), accumulation of lanosta-7,9(11), 24-trien-3α-o1-26-oic acid (ganoderic acid 24) (B) and total ganoderic acids (total GAs) (C) were evaluated. The means of three independent samples with standard deviations are presented. *p<0.05, **p<0.01, ***p<0.001 as compared with the control group.

The time course of GA induction by 4 mM aspirin was studied further. A reduction in fungal biomass was observed after 6 hr treatment with 4 mM aspirin (Figure 2A). Incubation of the fungal mycelium with 4 mM aspirin for 6 hr slightly enhanced total GA production, but had no effect on GA 24 production. Administration of aspirin for longer than 12 hr resulted in enhanced GA 24 production and total GAs production (Fig. 2B and 2C).

### Effect of fungal culture age on ganoderic acids induction

**Figure 3:**
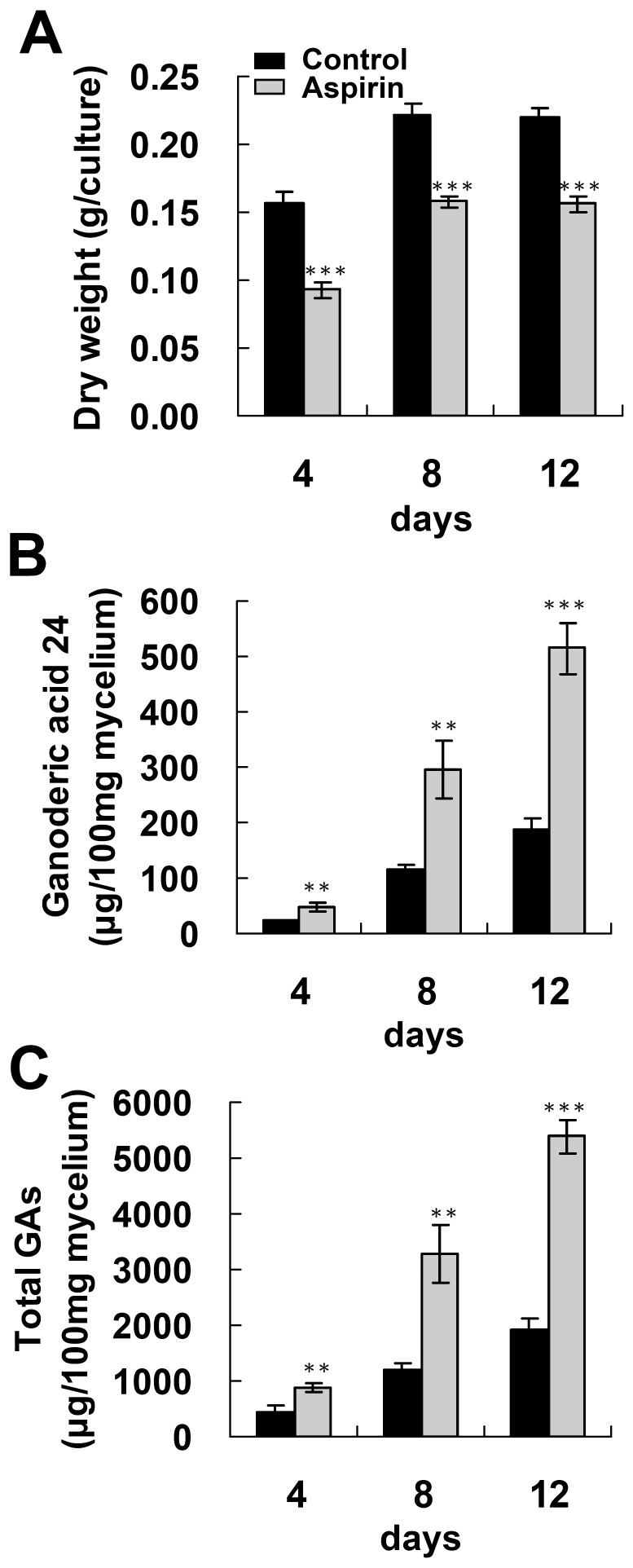
Effect of fungal culture age on ganoderic acids induction by aspirin in *Ganoderma lucidum*. Fungal mycelium cultured on PDA for 4–12 days was incubated with 4 mM aspirin for additional 1 day. Fungal biomass (A), lanosta-7,9(11), 24-trien-3α-o1-26-oic acid (ganoderic acid 24) production (B) and total ganoderic acids (total GAs) production (C) by *Ganoderma lucidum* were determined. The means of three independent samples with standard deviations are presented. *p<0.05, **p<0.01, ***p<0.001 as compared with the control group.

**Figure 4:**
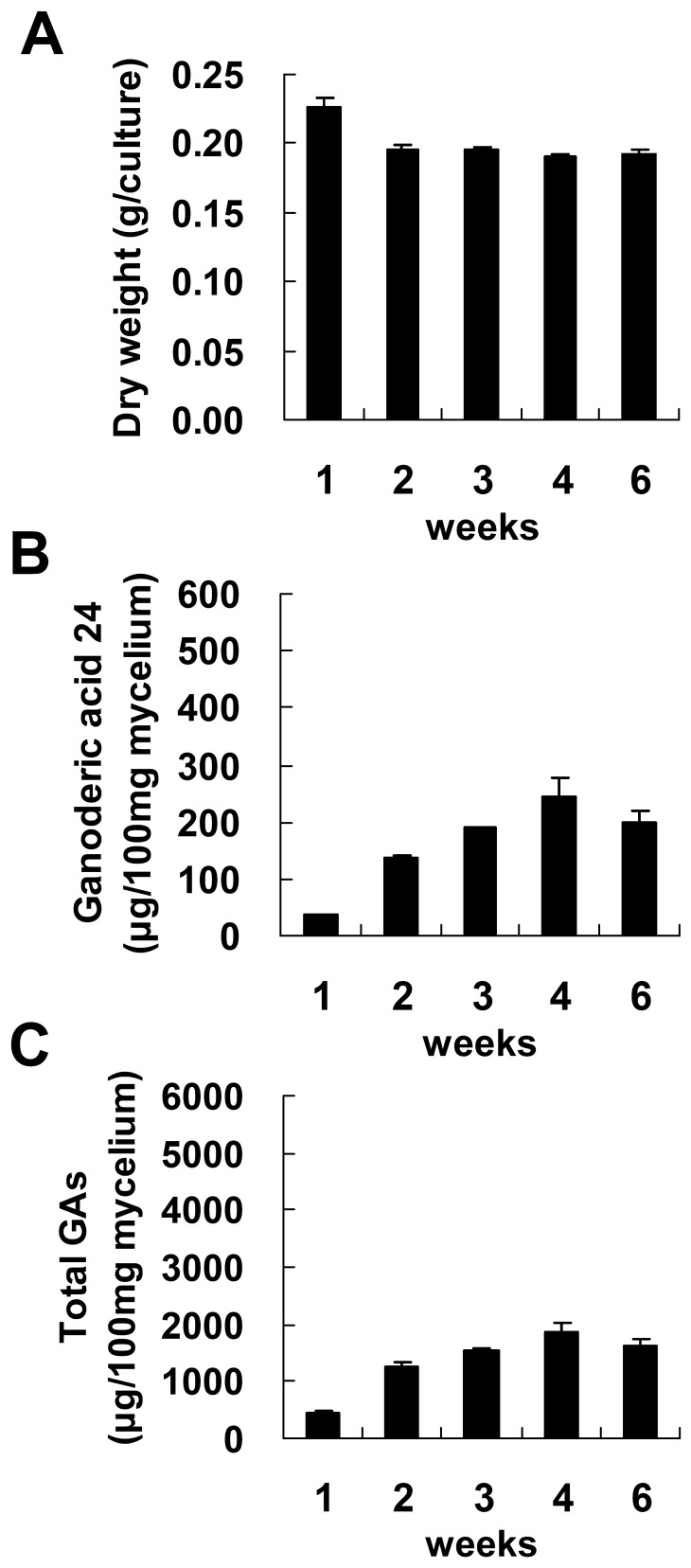
Time course of ganoderic acids and fungal biomass production of *Ganoderma lucidum* cultured on PDA. *Ganoderma lucidum* was cultured on potato dextrose agar (PDA) for 1 to 6 weeks. Fungal biomass (A), accumulation of lanosta-7,9(11), 24-trien-3α-o1-26-oic acid (ganoderic acid 24) (B) and total ganoderic acids (total GAs) (C) were evaluated. The means of three independent samples with standard deviations are presented.

To produce a maximum amount of GAs, fungal mycelium from different culture stages was tested for GA production after induction with aspirin. Fungal mycelium was cultured on PDA for 4 to 12 days and then was incubated with 4 mM aspirin for 1 day. As shown in Figure 3B and 3C, GA production by the control culture increased with the cultivation time. Furthermore, GA 24 production and total GA production were both significantly enhanced by aspirin across the various ages of fungal mycelium. Maximal GA24 production and total GA production were 515.2 and 5385 µg/100 mg dry weight (DW), respectively, and this was obtained using the 12-days old mycelium. This is a 2.7-fold and 2.8-fold increase compared with the control. In comparison, fungal mycelium cultured on regular PDA for 1.5 month only gave a maximum of 244.9 and 1857.2 µg/100 mg DW for GA 24 and total GAs, respectively (Figure 4). These results indicate that aspirin treatment is a powerful approach to triggering GA production over a short time.

### Aspirin induced apoptosis in the fungal cells of *G. lucidum*

**Figure 5:**
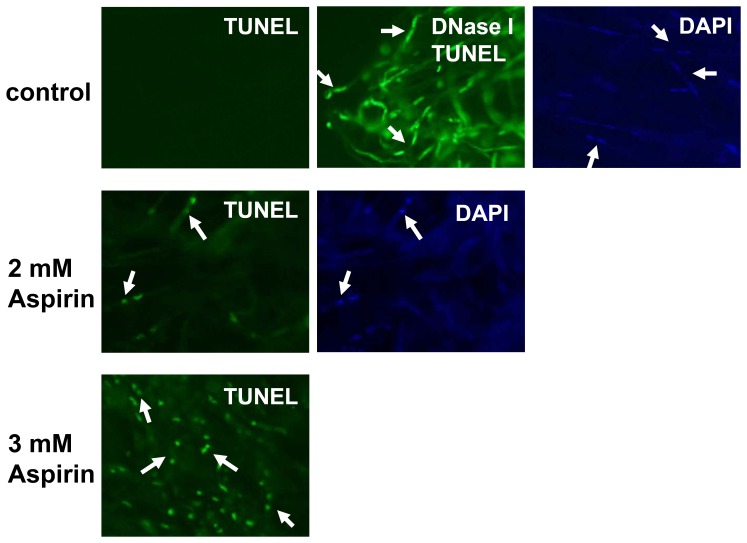
DNA fragmentation and nuclear morphology changes that occur in *Ganoderma lucidum* in response to aspirin. Fungal mycelium was incubated with aspirin followed by TUNEL assays and DAPI staining. To indicate the nuclear morphology of normal cells using TUNEL assay, fungal mycelium was pretreated with DNase I to induce DNA breaks and then interacted with the TUNEL reaction mixture. The arrows indicate two nuclei of each fungal cell in *G. lucidum*.

Fungal apoptosis displays certain characters; these include nuclear morphology changes and double-stranded DNA degradation. The latter can be examined by terminal deoxynucleotidyl transferase mediated dUPT nick end labeling (TUNEL) assays. Apoptosis of aspirin-treated fungal cells was evaluated by TUNEL assay and nuclear staining (Figure 5). Normal fungal cell did not show any fluorescent signal after TUNEL staining, indicating that the genomic DNA of normal cells was intact. To evaluate the nuclear morphology changes, 4′, 6-diamidino-2-phenylindole (DAPI) was used for nuclear staining. In addition, as a control, the chromosomal DNA of normal cells was pre-digested with DNase I and then assessed by TUNEL reaction mixture to show the nuclear morphology. Figure 5 showed nuclei observed in normal fungal cells. Fungal cells treated with 2 mM aspirin showed a few TUNEL positive cells and condensed nuclei were observed when the cells under the same condition were stained by either TUNEL or DAPI. A large number of TUNEL positive cells were observed in fungal cells incubated with 3 mM aspirin, and a condensed nuclear morphology was also presented (Figure 5). However, a high background level of DAPI staining was present in the fungal cells treated with 3 mM aspirin (data not shown). To our knowledge, this is the first report showing that aspirin is able to induce apoptosis in *G. lucidum*.

Our result suggests that GA biosynthesis occurs during cell apoptosis in *G. lucidum*. Previous studies have shown that secondary metabolite biosynthesis in fungi is coordinated with fungal development and is regulated by environment factors, including nutrition, pH, light and temperature [26]. Environmental and developmental cues then mediate secondary metabolites biosynthesis via a range of transcription factors and various signal transduction pathways such as heterotrimeric G-protein signaling, cAMP signaling, Ras family GTPase signaling and MAPK signaling [27], [28]. To the best of our knowledge, this study is the first to indicate that apoptosis signaling is correlated to fungal secondary metabolite biosynthesis. Other medicinal fungi such as *Inonotus obliquus*, *Poria cocos*, *Antrodia cinnamomea* and other *Ganoderma* species, have also been used as folk remedies for many centuries, and triterpenoids has been proved to be the functional components in these fungi [1], [29]–[31]. However, the regulation of triterpenoid biosynthesis in these fungi remains unknown. It is possible that apoptosis signaling regulates triterpenoid biosynthesis in these medicinal fungi too. In this context, apoptosis induction may have great practical value in the functional food industry where these fungi are used to produce functional components.

To further confirm the correlation of apoptosis signaling and GA biosynthesis in *G. lucidum*, various chemicals such as acetic acid and zinc chloride that have been shown to induce apoptosis in yeast [32] were incubated *G. lucidum*. Our results showed that incubating fungal mycelium with 20 mM acetic acid for 1 day increased GA 24 and total GAs production by 1.97- and 1.88-fold, respectively. Treatment of 5.4 mM ZnCl_2_ for 2 days also increased total GAs by 2.13-fold. This strongly supports the hypothesis that apoptosis signaling is involved in controlling GA biosynthesis.

In plants, the hypersensitive reaction, which can be regarded as a type of cell apoptosis, is induced by the presence of incompatible microbes or various elicitors from microbes. ROS production, the expression of defense genes, and antimicrobial secondary metabolite production are known to be induced during the hypersensitive reaction [33]. A few studies have indicated that fungal elicitors are able to induce cell apoptosis and the production of secondary metabolites, including taxol, artemisinin, and β-thujaplicin, in *Taxus chinensis*, *Artemisia annua*, and *Cupressus lusitanica*, respectively [34]–[36]. In addition to biotic inducers, abiotic stress has been widely used to increase plant secondary metabolite production [37]. However, whether abiotic stress induces secondary metabolites biosynthesis during cell apoptosis remains unknown. Recently studies have indicated that methyl jasmonate and ROS, which were previously used to enhance plants secondary metabolites production [38], also increased GA biosynthesis in *G. lucidum* [16], [19], [20]. These findings suggest that one or more common regulatory components may control secondary metabolite biosynthesis in fungi and plants. Thus, it is quite possible that apoptosis induction by abiotic stress may be an alternative approach to inducing plant secondary metabolite production.

### Effect of aspirin on expression of squalene synthase and lanosterol synthase mRNA

**Figure 6:**
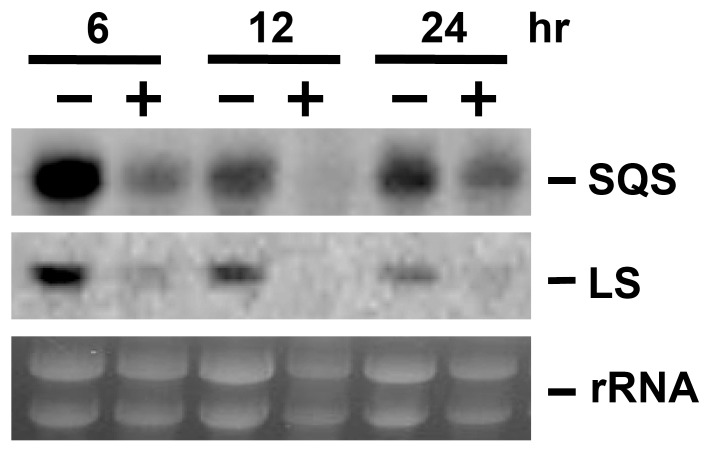
Transcription level of the squalene synthase (SQS) and lanosterol synthase (LS) in response to aspirin. Fungal mycelium of *Ganoderma lucidum* was incubated with 2 mM aspirin. Expression of SQS and LS coding region was determined by northern blotting. Gel stained with ethidium bromide was shown to indicate the relative loadings of the total RNA.

Both squalene synthase (SQS) and lanosterol synthase (LS) have been proposed to be involved in the biosynthesis of GAs. Gene expression of the SQS and LS in response to aspirin was assessed by Northern blotting analysis. The application of aspirin to *G. lucidum* cultures significantly reduced the levels of the SQS and LS gene transcripts (Figure 6). Our data also indicated that gene expression of the SQS and LS were reduced by acetic acid and zinc chloride (data not shown).

Xu et al. showed that GAs production and expression of SQS, LS, and 3-hydroxy-3-methylglutaryl CoA reductase (HMGR) was increased by static liquid culture as compared with shaking culture [13]. Methyl jasmonate and phenobarbital have also been demonstrated to increase GAs biosynthesis and expression of various biosynthetic genes [15], [16]. Over expression of HMGR in *G. lucidum* enhanced GA production indicating that HMGR play critical role for GA biosynthesis [39]. However, in this study, aspirin induced GAs production but reduced transcript of the LS and SQS. Our previous study has shown that high doses of ROS, which induce GAs biosynthesis, also reduce SQS and LS mRNA expression [19]. These findings support the idea that aspirin, as well as high doses of ROS, may up-regulate the GA biosynthetic genes down-stream of lanosterol biosynthesis [19]. However, the role of HMGR in apoptosis-induced GA biosynthesis is unknown.

### Effect of aspirin on reactive oxygen species production

**Figure 7:**
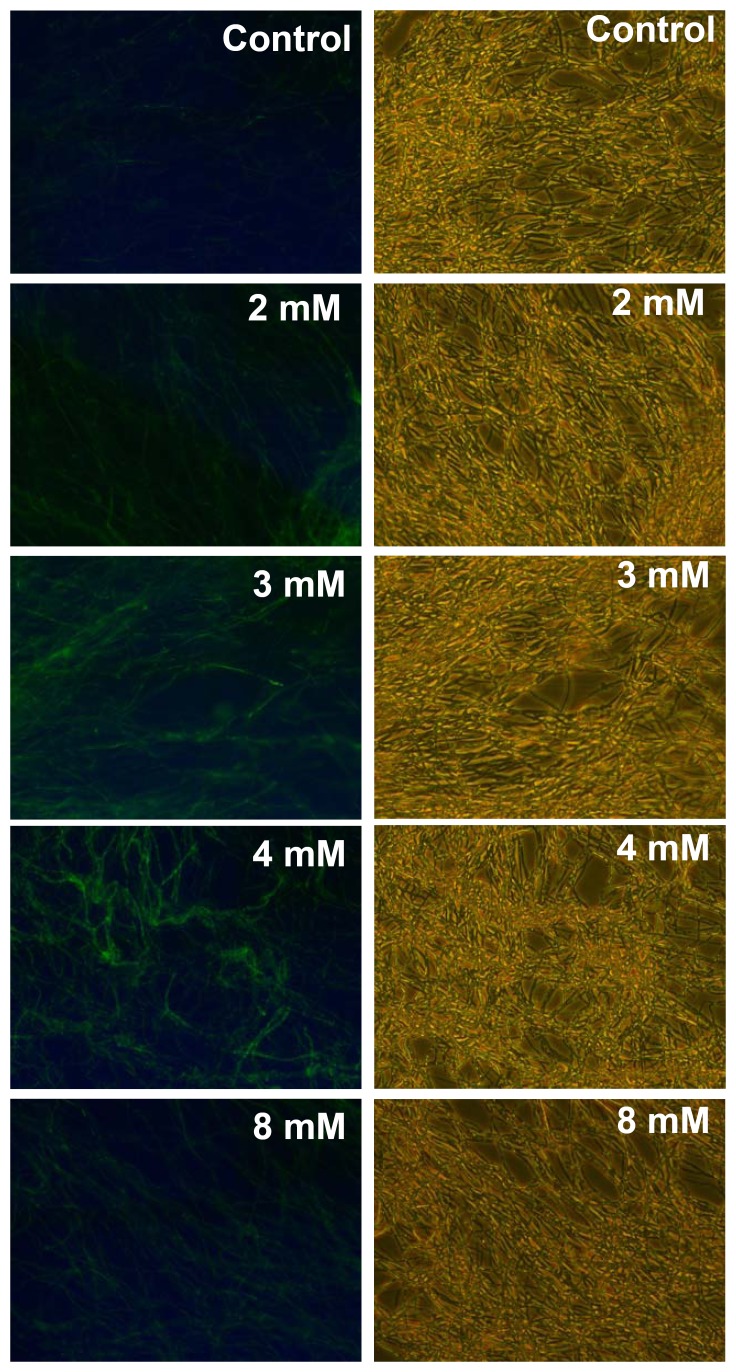
Reactive oxygen species production in *Ganoderma lucidum* incubated with aspirin. Fungal mycelium was pre-loaded with 2′,7′-dichlorofluorescin diacetate and then incubated with 2–8 mM aspirin for 4 hr.

Reactive oxygen species (ROS) has been proved to be an important regulator that is able to induce apoptosis. The putative role of ROS in aspirin-induced apoptosis in *G. lucidum* was evaluated. Fungal mycelium was incubated with aspirin and ROS production was evaluated using 2′,7′-dichlorofluorescin diacetate (DCFH-DA). No visible enhancement of fluorescence was detected when the fungal mycelium was treated with 0.5 mM aspirin (data not shown). As shown in Figure 7, enhanced ROS production could be observed in mycelium incubated with 2 mM aspirin and ROS was further increased as the aspirin concentration was increased to 4 mM. As compared with control, ROS accumulation of 4-, 11-, and 20-fold was obtained when fungal mycelium was incubated with 2, 3, and 4 mM aspirin, respectively.

In yeast, various signal molecules, including ROS and MAP kinase, have been shown to mediate apoptosis [21], [23]. Aspirin is known to induce oxidative stress and this causes mitochondrial dysfunction and apoptosis in human hepatoma HepG2 cells, indicating ROS play a crucial role in aspirin-induced apoptosis [25]. In contrast, Balzan R et al. suggested that ROS is probably not the primary signal involved in inducing apoptosis when a yeast mutant with a deficient mitochondrial manganese superoxide dismutase (MnSOD) was treated with aspirin [24]. In this study, ROS production was induced by treatment with 2 mM aspirin, at which concentration aspirin caused limited apoptosis. Furthermore, ROS increased in the presence of 3 mM aspirin, which also resulted in a high level of apoptosis. These findings suggest that ROS might be critical to aspirin-induced cell apoptosis in *G. lucidum*. Our results also showed that GAs production was significantly induced by 2 mM aspirin; however, under this circumstance, there was only a low level of double-stranded DNA degradation was detected. These results suggest that induction of GAs biosynthesis by aspirin might happen before the process of double-stranded DNA degradation which was triggered by higher concentration of aspirin.

### Effect of aspirin on the phosphorylation of mitogen activated protein (MAP) kinase

**Figure 8:**
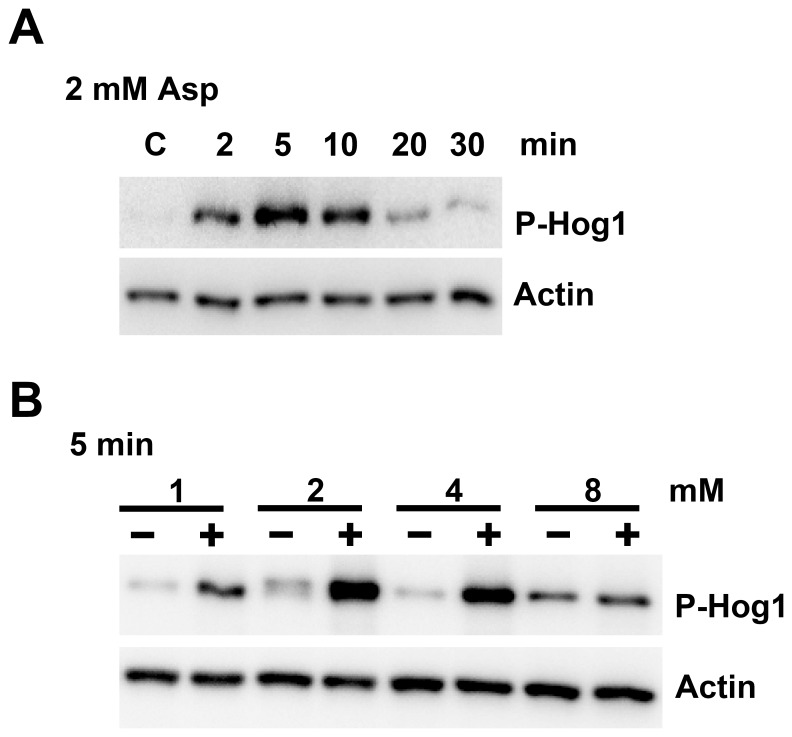
Phosphorylation of Hog-1 MAP kinases of *Ganoderma lucidum* in response to aspirin. (A) Fungal mycelium was incubated with 2 mM aspirin for 2–30 min. (B) Fungal mycelium was incubated with 1–8 mM aspirin for 5 min. Amount of actin detected by mouse anti-beta actin monoclonal antibody was used as the loading controls.

Hog-1 MAP kinase has been identified as a homolog of human p38 MAP kinase in fungi. To determine whether aspirin activates Hog-1 kinase in *G. lucidum*, antibody against the phosphorylated form of human P38 was used to detect the phosphorylation of Hog-1. After incubation of fungal mycelium with 2 mM aspirin, Hog-1 phosphorylation was observed after 2 min of aspirin treatment, and reached a maximum after 5 to 10 min incubation (Figure 8A). When different concentrations of aspirin were used, the phosphorylation signal was found to gradually increase up to 4 mM of aspirin (Figure 8B).

Intensive studies have been conducted to uncover how aspirin induce apoptosis in mammals and these have targeted p38 MAP kinase [40]. Phosphorylation of p38 MAP kinase has been shown to be significantly enhanced by aspirin in colorectal cancer cells. Application of a specific inhibitor to antagonize p38 kinase activation blocked aspirin-induced apoptosis, indicating p38 kinase mediated aspirin-induced apoptosis [41]. However, the MAP kinase signaling cascade has never been studied in fungi with respect to in aspirin-induced apoptosis. Hog1 has been shown to mediate the fungal stress resistance response and to be involved in sexual development, pathogenicity, and vegetative differentiation [42]–[45]. This study is the first to show aspirin induces Hog-1 phosphorylation, which indicates that Hog-1 may be involved in process of aspirin-induced fungal apoptosis. Our recent findings have also revealed that ROS and UV-B radiation are able to induce GA production and Hog-1 phosphorylation in *G. lucidum* [19]. These results suggest that Hog-1 may be associated with GA biosynthesis, which is known to be triggered by various environmental cues in *G. lucidum*. We are currently creating the genetic mutants of *G. lucidum* that are deficient in Hog-1 to clarify the gene's role in controlling GA biosynthesis in *G. lucidum*. In addition, the network controlling the various signaling pathways that regulate GA biosynthesis and apoptosis are under investigating by our group using both pharmacological and genetic approaches.

## Conclusions

Production and the biosynthetic regulation of secondary metabolites are important for the application of medicinal fungi and plants. Our results are the first findings to indicate that aspirin induces cell apoptosis in *G. lucidum* and that the induction of apoptosis coincides with GA biosynthesis. The findings presented here provided a novel and powerful approach to enhancing fungal secondary metabolite production, and potentially could be applied to other medicinal fungi and plants. Furthermore, our results indicate that ROS production and Hog-1 phosphorylation are induced by aspirin. This provides insights into the regulation of triterpenoid biosynthesis and the fungal apoptosis signaling cascade.
